# A Period of Grace: Suppression of Malignancy During Embryonic Life

**DOI:** 10.1002/cam4.72469

**Published:** 2026-11-11

**Authors:** Lena Einhorn, Ingemar Ernberg

**Affiliations:** ^1^ Department of Microbiology, Tumor and Cell Biology (MTC) Karolinska Institutet Stockholm Sweden

**Keywords:** antitumor soluble factors, carcinogenesis, embryonic cancer, tissue compliance, tumor suppression

## Abstract

It is conspicuously difficult to experimentally induce cancer in embryos, especially during organogenesis. Based on this, and a number of other aspects of tumorigenesis during intra‐uterine life, it has been hypothesized that there are factors present during embryogenesis that actively suppress the development of malignancy. In this paper we review the current evidence for this. We also review data on the effects of embryonic tissue extracts on cancer cells in vitro and in vivo, and discuss potential therapeutic implications.

## Plain Language Summary

It is conspicuously difficult to experimentally induce cancer in embryos, especially during organogenesis. It has been suggested that malignancy is actively suppressed during embryogenesis based on the following data: the difficulty to experimentally induce cancer in embryos; malignant cells transplanted to embryos often lose their malignant characteristics; the scarcity of cancer in newborns; spontaneous regression is not an uncommon finding in the very young and prenatal exposure to the atomic bombs led to 10 times fewer malignancies than post‐natal exposure. We also summarize published data on the effects of embryonic tissue extracts on cancer cells in vitro and in vivo.

## 1 Introduction

Experimental data clearly show that carcinogenesis is suppressed during embryogenesis. Most convincingly, attempts to induce cancer in early stage animal embryos by irradiation or by transplacental chemical carcinogenesis have largely been unsuccessful, even when exposed animals have been observed throughout their lifetimes. Instead, surviving offspring are born with a high rate of malformations. During later stages of gestation, animals rapidly become susceptible to carcinogenesis [1, 2, 3, 4], Figure 1.

**FIGURE 1:**
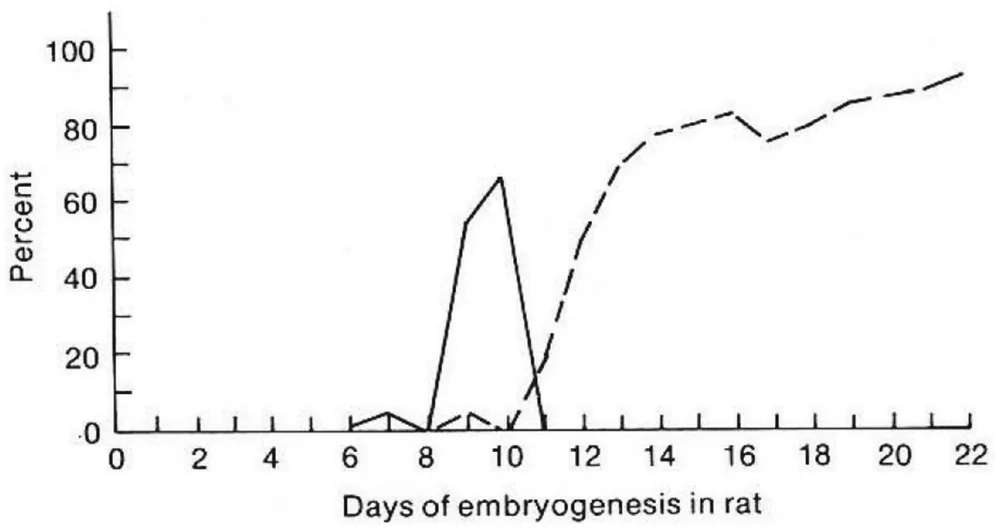
Effects of transplacental administration of ENU (20 mg/kg mother) in rats, at different stages of embryogenesis, with life‐long follow‐up. 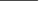 Teratogenic effect (hydro‐ and exencephaly, anophthalmos). 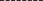 Carcinogenic effect (neurogenic tumors). Adapted from IARC Scientific Publications NO. 4, 1973. N.P. Napalkov. Published with permission from the editor.

Equally importantly, when malignant cells are transplanted to embryos, they frequently lose their malignant characteristics. They can even be integrated in the organism and participate in its normal development [5, 6, 7, 8, 9].

These data, along with other aspects of tumorigenesis in the embryo and fetus, led one of us to propose in 1983 that factors present during embryogenesis act to suppress the development of malignancies [10]. In 1991, a follow‐up article was published, focusing on the effects of prenatal irradiation on cancer development [11].

Although humans are very rarely born with cancer, or develop cancer within the first 28 days of life [12, 13], it is more challenging to document this tumor suppression in humans, due to an obvious lack of experimental data, but also to a number of statistical and epidemiological confounders. In particular, the lag time from cancer initiation to a detectable, manifest tumor, the variation in cell numbers and proliferating cells during the different stages of embryonic development, and generally a low cancer incidence during childhood and adolescence, contribute to this challenge. Nevertheless, available data in humans do not contradict, but rather support, the conclusion of cancer suppression during early embryonic life.

In summary, the hypothesis from 1983 was based on the following findings available at that time:

− Less than four in l00,000 humans are born with a cancer, or develop one during the first month of life [12, 13]. This should be compared to the 1%–4% of live‐born who have a diagnosable malformation at birth [14, 15].
− It is exceedingly rare to find a malignant tumor in spontaneously aborted embryos or fetuses [16, 17, 18].
− Although maternal cancer occurs in one out of 1000 pregnant women, there are very few reported cases of maternal cancer metastasizing to the embryo [19, 20, 21].
− A follow‐up at age 50 of survivors exposed prenatally and from birth to age 6 to the atomic bomb explosions in Hiroshima and Nagasaki showed a statistically significant dose‐related increase in solid tumors in both populations, but a 10‐fold greater risk among those exposed after birth, as compared to those exposed in utero [22].
− Attempts to induce cancer in early stage animal embryos by irradiation or by transplacental chemical carcinogenesis have been relatively unsuccessful, even when exposed animals have been observed throughout their lifetimes. Instead, surviving offspring are born with a high rate of malformations. During later stages of gestation, animals rapidly become susceptible to carcinogenesis [1, 2, 3, 4].
− The difficulties in experimentally inducing early stage malignancies in animals cannot be explained by a loss of the tumor‐prone population, through spontaneous abortion see [11].
− When removed from the embryonic environment, cells can easily be transformed, and thus the refractiveness to tumor development cannot primarily be explained at the single cell level [2].
− When malignant cells are transplanted to embryos, they frequently lose their malignant characteristics. They can even be integrated in the organism and participate in its normal development [5, 6, 7, 8, 9].
− In amphibia, which are capable of regenerating whole limbs, transplantation of a malignant tumor to a regenerating limb, or amputation of a limb distally of a chemically induced tumor, in many cases leads to differentiation and spontaneous regression of the tumor [23, 24, 25, 26, 27, 28]. This is a further indication that the intensity of the developmental processes correlates with the degree of protection against malignancy.
− So called embryonic tumors in humans arise mainly during the first 4 years of life and are comprised of immature tissues. However, in spite of their embryonic appearance, the most common embryonic tumors in humans—such as Wilms' tumor, neuroblastoma, medulloblastoma and retinoblastoma—arise in organs with an unusually late organogenesis [21]. These tissues partly retain their embryonic appearance until shortly before birth or even for some time after. It thus seems that in spite of the embryonic histology of these tumors, they probably develop at the end of gestation or after birth.
− The younger the child is when it develops an embryonic tumor (unlike leukemia), the better is generally the prognosis [29, 30, 31, 32, 33, 34, 35, 36, 37]. For instance, with regard to neuroblastoma, the survival rate in children less than 1 year old was at the time of publication determined to be around 35%, whereas in children diagnosed after age 2, only 5%–6% survived [32] Although survival rates have since then significantly improved, the striking difference in prognosis between younger and older children remains, in most cases also when the younger children have disseminated disease [38, 39, 40]. Also, adrenal neuroblastomas diagnosed in utero have an excellent prognosis, and often regress spontaneously [41].
− Unlike what is known for cancers in adults, spontaneous regression of embryonic tumors is not an uncommon finding [42]. These spontaneous regressions almost never occur after the age of 2 years. The modes of regression seem to be either necrosis of the tumor—as seen in retinoblastoma—or differentiation of the malignant tumor into a more highly differentiated benign tumor form—as seen in cases of spontaneous regression of neuroblastoma to benign ganglioneuroma [42, 43, 44]. Of 29 cases of spontaneously regressed neuroblastoma in one study, all were infants, and the mean age was 3 months [42].

The low frequency, and apparent inhibition, of cancer during early embryonic life thus appears to be attributable to the embryonic environment, rather than to intrinsic characteristics of embryonic cells. In addition, it is worth noting that the main mechanisms of embryonic tumor control—cell differentiation and cell death—are similar to those regulating normal embryonic development.

The conclusion that was then drawn was that cancer likely is controlled by mechanisms similar, or identical, to those that regulate normal development. Judging by the data, these mechanisms appear to be particularly pronounced and effective during organogenesis. The unusual sensitivity of the embryo and fetus to developmental disturbances, due to the high rate of growth and predominance of immature cells, seems to be counterbalanced by developmental‐regulatory processes which are most active during the most intense periods of growth and development. One could speculate that tumor development throughout life may be the result of an inability to retain the delicate balance between developmental activity and protection against developmental deviation.

The possible implications for alternative therapies against cancer, based on these insights, were obvious.

During the more than 40 years that have passed since the first of the two above‐mentioned articles were published, additional support for the protective milieu of the developing embryo has accumulated.

## 2 Additional Studies on Tumor Transplantation into an Embryonic Microenvironment

In the early 1970s, Ralph Brinster [5] and others [6, 7] demonstrated that when embryonal carcinoma cells from one strain of mouse were transferred into blastocysts of another strain and then implanted into the uterus of pseudo‐pregnant hosts, normal chimeric mice were produced, in which the originally malignant cells contributed to normal development.

Although this indicated that the embryonic environment acted to control, and reverse, the malignant state, the fact that the cancer cells were embryonic, and originally had been experimentally generated from grafted normal mouse embryos [45] limited a more extensive interpretation of the results.

Since then, however, in a number of studies the embryonic environment has been shown to induce documented reversal also when adult cancer cells have been transplanted into the embryo.

For example, Lee et al. showed that when human metastatic melanoma cells were transplanted into zebrafish blastula‐stage embryos, there was a true reversion of cancer cells to a non‐tumorigenic phenotype. The melanoma cells survived and divided, but did not form tumors. Rather than integrate into the host organs, however, they scattered throughout the embryo, residing in interstitial spaces [46]. Similar results were obtained in other studies, where human melanoma cells were transplanted into chicken [47, 48, 49] or mouse embryos.

In a study reminiscent of the chimeric mouse studies mentioned above—but using non‐embryonic tumors—Li et al. transferred nuclei from medulloblastoma tumors in mice to mouse enucleated oocytes, and then cloned blastocysts were transferred into pseudo‐pregnant females. Although no embryos developed to term, viable embryos were found until day eight, and there was no evidence of tumor formation [50]. Later, Hochedlinger et al. managed to produce chimeric mice from melanoma nuclei introduced into enucleated oocytes, which were used to generate embryonic stem cells, subsequently injected into blastocysts. Although the chimeric mice had a higher rate of malignancies than controls, the vast majority of their tissues were normal, again demonstrating the tumor‐reversing potency of the embryonic microenvironment [51].

Nevertheless, the timing of transplantation is relevant. Gootwine et al. injected myeloid leukemic cells into mouse embryos in utero at 10 days of gestation. This produced apparently healthy adult mice whose granulocytes contained a marker derived from the leukemic cells. However, when the leukemic cells were injected at other times of gestation, this was not achieved [9, 52], a result found also by other groups and for other tumors [53, 54, 55].

One possible interpretation of this is that the ability of the organism to inhibit the progression to cancer is greatest during those periods when the tissue in question is being most strongly regulated and organized, and in the vicinity of those developmental processes.

In general, and as before, the capacity of tumor cells to reverse into a non‐tumor phenotype in an embryonic environment has in different models been shown to decrease with advancing development [49, 56, 57, 58, 59].

## 3 Treatment of Tumors with Embryonic Stem Cells, Embryo Extracts, Oocyte Extracts, and Umbilical Cord Matrix Stem Cells: In Vitro Studies

Studies have thus clearly demonstrated that the early embryonic environment has the capacity to regulate, and reverse, the malignant state. The question is how.

To try to elucidate the mechanisms behind this period of grace—as well as to evaluate the potential for future therapies—in vitro studies have been conducted on the effects of embryonic factors and microenvironments on tumor cell lines.

For instance, Liu et al. co‐cultured a human uveal melanoma cell line with mouse embryonic stem cells, and found an inhibition of proliferation and tumorigenicity of the melanoma cells [60]. He et al. achieved similar results using a human glioblastoma cell line [61]. Ayuzawa et al. showed that also human umbilical cord matrix stem cells—which are easily accessible—inhibited growth of human breast cancer cells when cultured together [62].

Although some evidence suggests that tumor cell reversal by the embryonic stem cell microenvironment does rely on cell‐to‐cell contact [60, 63, 64], other studies, both in vitro and in vivo, have indicated that this may not necessarily be the case. Also, embryonic extracts, as well as diffusible substances, under certain conditions were shown to have the capacity to induce reversal and/or cell death.

In an early study, filtered extracts from 11 to 13 day chick embryos were added at different concentrations to A9 cells in soft agarose. At low concentrations of added embryo extract, the cells lived but could not form colonies, unlike the positive controls. At higher concentrations of embryo extract, the A9 cells died. Sephadex separation of the extract yielded a distinct inhibitory fraction (Einhorn and Mitrani, unpublished results).

Similar studies on malignant cell lines have been performed utilizing zebrafish embryo extracts and amphibian oocyte extracts. Biava et al. treated various tumor cell lines with factors extracted from zebrafish embryos at different stages. They found that a reduced tumor cell growth rate was observed when using factors extracted from all developmental stages, except the morula stage [65, 66, 67, 68, 69, 70]. Allegrucci et al. showed that breast cancer cells can be directly reprogrammed by amphibian oocyte extracts, with DNA demethylation and removal of repressive histone marks at the promoters of tumor suppressor genes [71].

Yang et al. found that when a human prostate cancer cell line was co‐cultured with human embryonic stem cells, the resulting supernatant, when removed, in itself decreased the viability of prostate cancer cells in a concentration‐dependent manner. This indicated a role of secreted factors, after stimulation with cancer cells [72]. Interestingly, Seno et al., in what looks like the reverse effect, found that conditioned medium from lung cancer or melanoma cells had the capacity to convert mouse embryonic stem cells into cancer stem cells [73].

## 4 Treatment of Tumors with Embryonic Extracts: In Vivo Studies

In vivo studies, where embryonic extracts have been used to try to modulate tumor formation in mice and rats, have been less unequivocal, and fewer studies have been published. Nevertheless, some groups have documented effects.

Liu et al. injected human uveal melanoma cells into nude mice. After approximately 2 weeks, the mice were injected in the tumor environment with either mouse embryonic stem cells, conditioned medium from mouse embryonic stem cells, or saline. After three treatment cycles, the tumors were examined. The tumors in the stem cell‐treated mice were much smaller than those treated with saline, whereas those treated with stem cell‐conditioned medium were only slightly smaller [60].

Ayuzawa et al. transplanted breast carcinoma cells into female mice. Starting 8 days later, the mice received three weekly injections of either saline or human umbilical cord matrix stem cells. All mice developed lung tumors. But in those that were treated with stem cells, tumor growth was significantly attenuated, and immunohistochemical analysis showed that the stem cells aggregated around the tumor tissue [58].

Biava et al. subcutaneously injected mouse lung carcinoma cells into female mice. Prior to injection, the tumor cells were mixed with homogenates of 9 day‐old mouse embryos, 9 day‐pregnant mouse uteri, non‐pregnant uteri or, as a control, liver. Only cells that were mixed with the homogenate of pregnant uteri failed to form tumors, whereas in all other cases tumors as well as metastases formed [74]. On the other hand, when homogenates of Drosophila embryonic tissues were used, a reduction in tumor size could be seen [75]. Positive results were also acquired when using stem cell differentiation stage factors from the zebrafish embryo [76].

Berger et al. found that when hepatoma cells were injected into syngeneic rats, pretreatment of the cancer cells with mouse embryo cell nuclei extracts increased survival of the rats [77].

Yang et al. injected prostate cancer cells subcutaneously into mice. They found that after tumors formed, the subsequent injection around the tumors with supernatant harvested after co‐culture of the prostate cancer cells with human embryonic stem cells inhibited growth of the tumors. This was unlike the result when saline or the supernatant from cultured embryonic stem cells alone was injected [72].

Bischof et al. found that cell‐free extracellular matrix (ECM) isolated from embryonic mouse mammary mesenchyme also can have a tumor inhibitory effect. When injected into mammary tumors in mice, the ECM‐treated animals displayed tumors 44% smaller than the controls [78].

How significant these findings are is difficult to determine, due to the publication bias towards successful experiments.

## 5 Treatment of Tumors with Embryonic Extracts: Clinical Studies

In addition to the experimental in vivo studies, there are a few preliminary clinical trials that have been conducted, where late‐stage malignancies in humans were treated with embryonic extracts.

Livraghi et al. conducted a randomized controlled study on 179 patients with intermediate‐advanced hepatocellular carcinoma, unresponsive to conventional treatment. Patients were randomly assigned to sublingually receive low molecular weight extracts from zebra fish embryos at the 50% epiboly stage, or, as a control, only conservative treatment. In the group treated with the embryo extract, the authors reported a 20% rate of partial and, in some cases, complete regression, and a 16% rate of disease stabilization after 6 months, whereas the control group had a 94% progression of disease. On the basis of the early results, randomization was discontinued [79]. In a follow‐up study, the authors reported confirmation of these results [80, 81].

Furthermore, members of the same research team conducted a pilot study on heavily pretreated patients with advanced and relapsing colon cancer. The patients were included in a trial to receive salvage treatment with the multi‐kinase inhibitor Regorafenib. A group of 12 patients was further randomly assigned treatment with a formula containing zebrafish embryo extracts, in addition to Regorafenib. At 12 months, the authors reported a 33% survival rate among those who received Regorafenib alone, and a 75% survival rate among those receiving the combination treatment [70, 82]. This was in line with their in vitro findings that zebrafish embryo extracts could enhance the effect of chemotherapeutic drugs on colon cancer cells [69], as well as on breast cancer cells [83].

However, in another randomized trial, conducted by another group, the formula containing zebrafish embryo extracts was compared with the hormone megestrol in the treatment of 61 patients with advanced hepatocellular carcinoma. And in this study, megestrol treatment was found to provide better long‐term survival than the zebrafish extract [84].

Yet another group used the zebrafish embryo extract in conjunction with the kinase inhibitor Sorafenib in the treatment of a hepatocellular carcinoma which then underwent complete regression [85]. The relative contribution of each compound to the end result was not determined.

In conclusion, these clinical results must be regarded as very preliminary, and not yet thoroughly assessed or validated.

It is worth noting, however, that at least with regard to one adult cancer, the highly malignant acute promyelocytic leukemia, clinical reversion to a benign state through differentiation and apoptosis has been clearly and consistently demonstrated, after treatment with all‐trans retinoic acid and arsenic trioxide. Although in that case the management does not employ factors from the microenvironment, it further demonstrates the capacity for at least some malignancies to be forcibly differentiated to normality, also in a clinical setting [86].

## 6 Mechanisms of Tumor Reversion in the Embryonic Environment

A considerable amount of work has gone into elucidating the mechanisms behind the demonstrated tumor inhibitory effects of the embryonic microenvironment. To begin with, it has long been clear that cancer cells share distinctive features with embryonic cells—a multipotent and plastic phenotype, unrestrained proliferation, expression of embryonic proteins, epithelial‐mesenchymal transitions, etc. In addition, it is clear that the embryonic microenvironment, at specific stages, can reprogram tumor cells to a normal phenotype, without damaging them [46], something which shows that cancer development can be a reversible process and potentially could be of enormous therapeutic significance.

Studies on the processes of tumor cell reversion, after treatment employing the embryonic microenvironment, have demonstrated that the malignant cells can regain what appears to be fully normal characteristics: a reinstatement of normal molecular pathways, cell cycle arrest, normal morphology, normal cell‐to‐cell adhesions, a down‐regulation of epithelial‐mesenchymal transitions, a rewiring of the cytoskeletal architecture, a reversal of DNA methylation, removal of repressive histone marks at the promoters of tumor suppressor genes, and other molecular and morphological aspects of normal cell development [87].

Nevertheless, the precise mechanisms through which the embryonic environment actually steers the developing organism on its perilous journey from pluripotency to normal, differentiated tissues and organs, and thwarts the potential progression to malignancy are still partly shrouded in mystery. Although in vitro studies have shown that cell‐to‐cell or tissue‐to‐tissue contact between embryonic cells and cancer cells is a beneficial and sometimes necessary feature of reversion [60, 64], there also seem to be ways to at least partially bypass this. For example, Yang et al. found that the co‐culture of a prostate cancer cell line with embryonic stem cells produced secretory factors that in themselves later could elicit reversion [72]. In the above mentioned small clinical trials on the treatment of advanced hepatocellular and colon cancers with embryonic zebrafish low molecular weight extracts, there was obviously no cell‐to‐cell contact. At the same time, the authors themselves write: “it is quite hard to fathom why those patients achieved such a remarkable response. No single active component has been hitherto identified within the crude protein extract from the Zebrafish embryo, or from any other conditioned embryo milieu, that could explain the observed pharmacological effect. An extensive search for those anti‐cancer factors has been performed. Yet, no conclusive data have been provided and the identification of embryo‐related anticancer molecular effectors is still a matter of intensive research” [82].

## 7 Discussion

It is in this context important to consider some overall aspects of tumorigenesis throughout life: It must be recognized that cancer primarily is a disease of aging. In humans, diagnosis of cancer is relatively rare until 45–50 years of age, when the incidence rises sharply with a peak around 75–80 years, after which it declines again [88]. This is usually explained by the most established mechanism of the disease, that is an evolutionary‐like process at the cell population level, driven by an accumulation of mutations in driver genes, leading to a selection of cell clones which form the basis of malignancy. In common human adult cancers, like breast cancer, prostate cancer and colon cancer, it has been concluded that some 10 or more critical mutations in driver and suppressor genes are needed for the establishment of a clinically manifest tumor [89]. To accumulate these mutations obviously takes time, sometimes estimated to be several decades. One interesting observation, however, is that after the age of 80–85, cancer incidence drops dramatically, and virtually no centennials get malignancies. The explanations vary from statistical errors in recording the incidence in the very old, to the assumption that those with a proneness to cancer have already been diagnosed with a malignancy by the age of 85, to perhaps a low degree of tissue renewal and cell proliferation in the elderly [90, 91, 92, 93]. But no consensus has been reached on this interesting phenomenon.

In newborns and young children, the panorama of cancer is different than in adults. Tumors in the young seem to depend on fewer mutations and are further affected by a whole range of inherited germ line mutations, which increase the cancer risk. The difference in cancer incidence between young children and adults at peak incidence age is several 100‐fold [88]. In spite of this dramatic difference, cancer in the newborn is a further fivefold lower than in children 0–5 years old [94, 95, 96], and close to non‐existent in spontaneously aborted embryos or fetuses [16, 17, 18].

With regard to malignancy, embryonic life is truly a period of grace.

It could of course be argued that this period of grace in the early embryo is a function of not having had time to accumulate enough mutations. But the above mentioned findings—that cancer cells transplanted to the embryo lose their malignant phenotype, that maternal cancer does not metastasize to the embryo, that embryonic tumors have the capacity to spontaneously regress, that attempts to induce cancer in early stage animal embryos have been so unsuccessful even when exposed animals have been observed throughout their lifetimes—all speak against the embryonic resistance to tumorigenesis being due to a paucity of necessary mutations or a lack of time for tumor development.

Nevertheless, in humans, the data on embryonic tumor initiation, and much later malignant transformation, are not entirely clear‐cut. Certain types of maternal carcinogenic exposure have been shown to somewhat increase the risk of much later cancer development in the offspring. The most well‐studied example is maternal use of diethylstilbestrol during pregnancy, mainly in the 1950s and 1960s, leading to the development of clear‐cell adenocarcinoma of the vagina in the daughters, usually at age 14–23. The adenocarcinoma incidence was, however, very low, estimated at 0.14–1.4/1000 [97, 98], whereas the incidence of other vaginal epithelial changes, such as adenosis, was as high as 34% [99]. Importantly, in humans, organogenesis—which seems to be the most tumor resistant period—mainly takes place during the first 2 months of intra‐uterine life, and the mothers had as a rule been treated with diethylstilbestrol throughout pregnancy.

With regard to the effects of human intra‐uterine exposure to radiation, the largest and best studied cohort consists of those exposed in utero to the atomic bomb explosions in Hiroshima and Nagasaki, in 1945. A follow‐up at age 50 of survivors exposed prenatally (2452 individuals) and from birth to age six (15,388 individuals) showed a statistically significant dose‐related increase in solid tumors in both populations, but a 10‐fold greater risk among those exposed after birth, as compared to those exposed in utero. This again demonstrates that carcinogenic insults during intra‐uterine life have more limited effects than those after birth. In this study, no statistically significant difference in risk was detected for different trimester exposures [22]. Another study of those exposed in utero followed them up to age 67, focusing on causes of death [100].

Much has also been written about the potential multigenerational effects of carcinogens, and about multigenerational transmission of hereditary germ cell mutations [101]. Perhaps the best example of this is the germ‐line mutation predisposing for hereditary retinoblastoma, which typically gives rise to retinal malignancies during the first year of life, with an 80%–90% penetrance. On the face of it, the survival of these tumor‐prone cells through intra‐uterine life would seem to argue against the tumor‐inhibitory effects of the embryonic environment. In addition, mothers with several retinoblastoma children do not have a higher than normal rate of miscarriage, so the mutation‐carrying population is not diminished through spontaneous abortion [102]. The reasonable conclusion, however, is that a genetic aberration alone—without the expression of the malignant, or otherwise abnormal, phenotype—is not necessarily enough to trigger tumor inhibition. However, it cannot be excluded that there is more than one mechanism.

Is the suppression of tumor development during organogenesis due to reversion or elimination of already transformed cells or does it take place at an earlier stage, that is it due to protection against initiation of cancer at the cellular level (e.g., protection against the first initiating mutation)?

As we have demonstrated, most experimental data speak to an effect at the tissue level due to the presence of tumor‐suppressive factors in the embryonic environment. However, there is data supporting a protective effect against mutations also at the cellular level, data indicating that somatic stem cells in tissues with constant active renewal are relatively protected against induction of mutagenesis by radiation and other mutagens. This effect has been studied especially in model systems of hair follicle stem cells and in intestinal stem cells [103, 104, 105]. It seems primarily to be due to an enhanced DNA‐repair function.

It should be noted that in certain organisms, demonstrable tumor suppression can occur also in adult life. As mentioned above, in adult amphibia, transplantation of a malignant tumor to a regenerating limb, or amputation of a limb distally of a chemically induced tumor, in many cases leads to differentiation and spontaneous regression of the tumor [23, 24, 25, 26, 27, 28]. In line with this, Gersch noted that animal species with a high capacity for regeneration have a comparatively low rate of tumors [106]. But in some species with a low rate of tumors there may be other mechanisms at hand. In the naked mole rat, the suppression of malignancy is considered to be due to unusually large molecules of hyaluronic acid in the connective tissues [107]. Alternative explanations have been based on DNA repair or immune suppression [108, 109]. In large, cell‐rich animals like elephants and whales, there also seems to be a surprisingly low frequency of tumors. In elephants this has been related to a strong gene copy amplification of the tumor suppressor gene p53, whereas in whales, there seems to be a general amplification of tumor suppressor genes [110, 111, 112, 113].

Among other noteworthy observations is the very low incidence of cancer in the small intestine of adult humans, despite a very high proliferation rate, as compared to the high incidence of cancer in the colon, which also has a high rate of cell turnover [114]. An effort to explain this has been the vast difference in the load of microbes between the small intestine and the colon. But also in this case, the exact mechanism is not evident.

To understand how morphogenesis works and anatomical homeostasis is maintained, the connection between the genetically determined cellular hardware and the physiological software of multilevel organization needs to be clarified [115]. With this perspective it is tempting to consider that the large‐scale process resulting in cancer is not just a single‐cell problem but a disorder of cellular coordination at the system level [116].

The ubiquitous electric fields created by ion channels around cells and at the tissue level are one emerging mechanism which has been suggested to operate at this system level. Bioelectrically‐mediated information flows bind cells leading to anatomical outcomes, and this represents an attractive control system of morphogenesis.

Cancer can be viewed as the consequence of a breakdown of the communication between cells that enable them to join into networks which work towards large‐scale morphogenesis [117].

## 8 Concluding Remarks

Already in the 1800s, Virchow [118], and later Cohnheim [119, 120], and Durante [121], suggested that malignancies represent an aberrant form of cellular development and that cancers arise from embryonal tissues remaining in the adult organism. In the 1930s, Waddington [122] and Needham [123] hypothesized that cancer represents an escape from morphogenetic fields, similar to those that guide embryonic development.

In the 1970s, several groups could demonstrate that when teratocarcinoma cells from one mouse strain were transferred into blastocysts of another strain, and then implanted into the uteri of pseudo‐pregnant hosts, normal chimeric mice were produced, in which the originally malignant cells contributed to normal development [5, 6, 7].

The fact that the teratocarcinoma cells in themselves were embryonic, and originally had been experimentally generated from grafted normal mouse embryos, limited a wider interpretation of the results. But the finding that other studies scattered throughout the literature did, in fact, provide indications of an embryonic period of grace—a very low incidence of newborns with cancer, difficulty to experimentally induce cancer in early stage embryos, the findings that embryonic cells, when removed from the embryonic environment, can easily be transformed, etc. in 1983 prompted one of us to publish the hypothesis that there are factors present during embryonic life, and especially during the period of organogenesis, that actively inhibit the development of cancer [10].

In the ensuing decades, further studies have strengthened the conclusion that components in the embryonic environment—components whose primary function is to steer the developing organism from a state of pluripotency and unconstrained proliferation to one of differentiated and specialized tissues and organs—also function as inhibitors of aberrant development, in the form of malignancies. Numerous studies have shown that the early embryo, in particular during organogenesis, reacts to malignant influences—be they chemical carcinogens, radiation, or transplanted malignant cells—by either forcibly differentiating the affected cells or inducing cell death. Later in development, this capacity is radically diminished. The fact that the same carcinogenic agents which during later fetal life and after birth in surviving offspring will induce a large number of malignancies, instead, when administered during organogenesis, will produce malformations but no malignancies, is a palpable demonstration of a strongly protective morphogenetic field early in development [1, 2, 3, 4, 46, 47, 48, 49].

Morphogenetic fields are of course present throughout life, and, as Bissell and others have demonstrated, the microenvironment has a decisive effect on tissue renewal as well as tumor development long after birth [124]. Genetic alterations are far from being the only determining factors in tumorigenesis; the environment matters. In some circumstances, the environment can even induce cancer. Recombining normal epithelium with carcinogen‐treated stroma can lead to malignant transformation of the epithelium [125, 126].

But the regulation of normal, and aberrant, growth is never as strong and potent as during the organism's early development. And never in life is the capacity for actual tumor reversal so pronounced.

The crucial question, and challenge, is if the demonstrated boosted prevention of neoplastic processes in the embryonic environment ultimately can have clinical applications.

The fact that embryonic factors can reprogram malignant cells into harmless ones, without necessarily killing them, would, if these factors and mechanisms could be identified and harnessed, be of enormous potential clinical benefit.

## Author Contributions

**Lena Einhorn:** writing – original draft, conceptualization, investigation, methodology, validation, visualization, data curation. **Ingemar Ernberg:** funding acquisition, writing – review and editing, project administration, supervision, resources.

## Funding

This work was supported by grants from the Swedish Cancer Society (no. 2222 72Pj 01H). and Radiumhemmets Forskningsfonder (no. 211011).

## Conflicts of Interest

The authors declare no conflicts of interest.

## Data Availability

The data that support the findings of this study are available from the corresponding author upon reasonable request.
